# Expression of Trace Amine-Associated Receptors in the Murine and Human Hippocampus Based on Public Transcriptomic Data

**DOI:** 10.3390/cells11111813

**Published:** 2022-06-01

**Authors:** Nataliia V. Katolikova, Anastasia N. Vaganova, Evgeniya V. Efimova, Raul R. Gainetdinov

**Affiliations:** 1 Institute of Translational Biomedicine, Saint-Petersburg State University, 199034 Saint-Petersburg, Russia; e.v.efimova@mail.ru (E.V.E.); gainetdinov.raul@gmail.com (R.R.G.); 2 Saint-Petersburg State University Hospital, Saint-Petersburg State University, 199034 Saint-Petersburg, Russia

**Keywords:** trace amine-associated receptors (TAARs), TAAR1, TAAR2, TAAR5, TAAR6, TAAR8, TAAR9, hippocampus, gyrus dentatus, adult neurogenesis

## Abstract

Hippocampus is one of the neurogenic zones where adult neurogenesis takes place. This process is quite complex and has a multicomponent regulation. A family of G protein-coupled trace amine-associated receptors (TAARs) was discovered only in 2001, and most of them (TAAR2-TAAR9) were primarily considered olfactory. Recent studies have shown, however, that they are also expressed in the mouse brain, particularly in limbic formations, and can play a role in the regulation of emotional behaviors. The observations in knockout mice indicate that at least two members of the family, TAAR2 and TAAR5, have an impact on the regulation of adult neurogenesis. In the present study, we analyzed the expression of TAARs in the murine and human hippocampus using public RNAseq datasets. Our results indicate a low but detectable level of certain TAARs expression in the hippocampal cells in selected high-quality transcriptomic datasets from both mouse and human samples. At the same time, we observed the difference between humans, where TAAR6 expression was the highest, and murine samples, where TAAR1, TAAR2, TAAR3, TAAR4 and TAAR5 are more pronouncedly expressed. These observations provide further support to the data gained in knockout mice, indicating a role of TAARs in the regulation of adult neurogenesis in the hippocampus.

## 1. Introduction

Adult neurogenesis was first described in 1960s [1]. It is believed that the process of formation of new neurons goes on throughout the whole life period, but its intensity significantly decreases with age [2]. Neurogenesis in the adult brain takes place in neurogenic niches, which are located in the ventricular–subventricular zone (V-SVZ) and in the subgranular zone (SGZ) of the gyrus dentatus (DG) [3,4]. DG and hippocampus are the parts of the hippocampal formation, which plays an essential role in learning and memory.

**Figure 1:**
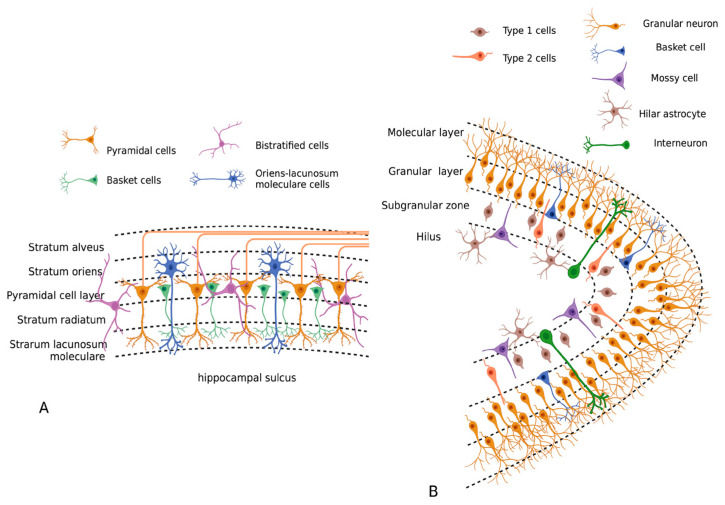
Structure of hippocampus (**A**) and gyrus dentatus (**B**). Created in BioRender.com.

DG has C-shape and, at the cellular level, has several distinct layers: the molecular, granule cell layers, subgranular zone and hilus (polymorphic layer) (Figure 1B). The molecular layer is made from dendrites of the dentate granule cells with a small number of intermediate neurons. The granular layer comprises mainly granule cells. The subgranular zone contains neural progenitor cells, which differentiate and become granular neurons. The border between the granular and subgranular zones is rich with pyramidal basket cells, and the hilus is predominantly represented by mossy cells.

The hippocampus also has C-shape and is divided into three different areas based upon histology features: CA1, CA2 and CA3. All of them have five cell layers (Figure 1A). The stratum alveus contains the alvear pathway and is represented by the oriens-lacunosum moleculare cells and basal dendrites of pyramidal cells. The pyramidal cell layer contains pyramidal cells and a number of basket cells, and the stratum radiatum with stratum lacunosum-moleculare contains a perforant pathway composed of the apical dendrites of pyramidal cells and hippocampal afferent fibers coming from the entorhinal cortex.

During adult neurogenesis, the neuronal stem cells became fully maturated neurons. In DG, it leads to the formation of only one type of neurons—granule cells [5]. The process starts from radial glia-like precursor cells (type 1 cells), which give rise to intermediate progenitor cells, type 2a and then type 2b. As the next type, 2b cells became type 3 cells. These cells became immature neurons, and after maturation, they fully integrate into the existing network, becoming indistinguishable from the older granule cells.

The regulation of adult neurogenesis is also very complex. The most central among them are transcription factors with their receptors, a large number of which are G protein-coupled receptors (GPCRs), their signaling cascades, products of lipid metabolism [6] and metabolic components, including the status of oxidative phosphorylation [7].

Biogenic monoamine neurotransmitters, such as norepinephrine, dopamine and serotonin (5-HT), are known to be involved in the adult neurogenesis regulation via activation of their respective GPCRs [8,9]. The least studied group of monoaminergic GPCRs, trace amine-associated receptors (TAARs), were identified in 2001 [10,11]. Trace amines are the class of endogenous biogenic amines represented by β-phenylethylamine, tyramine, tryptamine and many other products of decarboxylation of amino acids, including cadaverine and putrescine [12]. Nine subtypes of TAAR genes are identified in mammals. In humans, six subtypes are functional—TAAR1, TAAR2, TAAR5, TAAR6, TAAR8 and TAAR9, with three receptor genes described as pseudogenes. In mice, all six subtypes of the receptors are functional, with six TAAR7 receptors (one of them is a pseudogene) and three TAAR8 receptors [12]. So far, a neuronal function of the TAAR1 receptor was best characterized while all other TAARs were mostly considered as olfactory receptors sensing innate odors, including pheromones and the smell of decomposed tissue [13]. However, recently the presence of TAAR2 and TAAR5 receptors in the mouse brain was shown [14,15,16,17]. By using TAAR2 and TAAR5 knockout mice expressing beta-galactosidase to map the pattern of their expression, we observed that TAAR2 and TAAR5 are expressed in several brain regions, predominantly in the limbic areas, including the hippocampus [16,17].

The studies performed in knockout mice indicated that TAAR2 and TAAR5 can modulate dopamine and serotonin systems and are involved in the regulation of emotions [16,17]. Recent discoveries have shown that TAAR2 and TAAR5 can also be involved in the regulation of neurogenesis. Animals lacking TAAR2 or TAAR5 genes have an increased number of dopaminergic neurons in the substantia nigra, an increased expression of brain-derived neurotrophic factor (BDNF) or glial cell line-derived neurotrophic factor (GDNF), respectively, in the striatum, and an increased number of doublecortin-positive PCNA-positive neuroprogenitor cells in the SGZ of DG [14,15,16]. The involvement of the TAARs in emotional regulation and adult neurogenesis makes them an interesting and prospective target in pharmacotherapy. In this study, we focused on the bioinformatic analysis of available transcriptomic data on the TAARs expression levels in the hippocampus of mice and humans.

## 2. Materials and Methods

### 2.1. Data Collection and Inclusion Criteria for Datasets

RNA sequencing data were searched in the National Center of Biotechnology Information (NCBI) Gene Expression Omnibus (GEO) [18] for the terms “hippocampus”, “hippocampal”, “CA1”, “CA2”, “CA3”, “gyrus dentatus” and “dentate gyrus” through 1 February 2022. Each GEO dataset should meet the following criteria: (1) complete TAARs expression data in raw counts, FPKM or TPM; (2) clear explanation of sample origin (i.e., whole hippocampus or a specific part of it); (3) because of low TAARs mRNA transcription levels, only datasets consisting of samples with the minimum number of reads in SRA files > 50 million reads were selected. After excluding irrelevant datasets, four datasets generated by RNAseq for the entire hippocampus and one dataset generated by RNAseq of ribosome-associated mRNA in CA1 pyramidal cells were included in the review (Table 1).

To identify the cell source of TAAR’s mRNA in the hippocampus, the Single Cell Portal database (available at https://singlecell.broadinstitute.org/single_cell, accessed on 15 February 2022) was searched for the term “hippocampus”. When all records without data on TAARs expression were excluded, the only remaining record, SCP1 “Study: Single nucleus RNA-seq of cell diversity in the adult mouse hippocampus (sNuc-Seq)”, was included in the review (Table 1). The distribution of TAARs expression in the cell populations was analyzed and visualized using the Single Cell Portal interface.

In addition, the single-cell transcriptomic dataset GSE75386 with a sequencing depth of 1–16 million reads per cell was also analyzed in this study.

### 2.2. Raw Data Normalization and Analysis

For uniform estimation of the expression levels, all data were TPM-normalized. TPM values above the threshold level of 0.5 were considered positive. The distribution of TPM-normalized expression levels in hippocampus samples was analyzed and visualized by the beeswarm R package.

The single-cell dataset SCP1 “Study: Single nucleus RNA-seq of cell diversity in the adult mouse hippocampus (sNuc-Seq)” is represented by TPM-normalized values. It was analyzed as it stands by the Single Cell Portal interactive interface.

### 2.3. Genotype-Tissue Expression (GTEx) Data

GTEx transcriptomic data were obtained from dbGaP accession number phs000424.v8.p2 on 17 March 2022. Data were visualized by GTEx Portal interactive interface available at https://www.gtexportal.org/home/ (accessed on 17 March 2022).

### 2.4. Statistical Analyses

Since the data from GEO data included in the overview were presented in raw counts, the expression values were normalized using the trimmed mean of M-values (TMM) method of the edgeR package [19] to avoid batch effects. Subsequently, gene expression data were normalized using the voom function in limma [20]. Differentially expressed genes were identified by the empirical Bayes test using the limma package [20]. The normalized data were fed into the lmFit and eBayes functions.

## 3. Results

### 3.1. TAARs mRNA Expression in Whole Hippocampal Specimens

The range of transcriptomic datasets representing the diversity of expression patterns in the hippocampus was narrowed down to the most deeply sequenced samples analyzed in mouse studies. No datasets consisting of human data met the criteria for inclusion in the review. As reviewed datasets consist of control groups and matched experimental groups, differential expression analysis was performed in all datasets to estimate the possible difference in TAARs expression between untreated animals and animals treated under the experimental conditions or maintained under atypical experimental circumstances. The differential expression analysis revealed no influence on the size of the animals’ social network, maternal exposure to urban particulate matter, sleep deprivation or long-term potentiation of the hippocampal slices in vitro after sampling the TAARs expression pattern (*p* > 0.05).

Since no significant differences in TAARs expression could be detected between control and experimental animals, the groups were merged for further analysis so that, in total, TAARs expression was estimated in 79 hippocampal whole tissue samples. The group studied consisted of 50 males and 29 females. In total, 50 animals were 2–4 months old C57BL/6 mice, and the other 29 animals were wild house mice (*Mus musculus domesticus*) whose age is uncertain.

**Figure 2:**
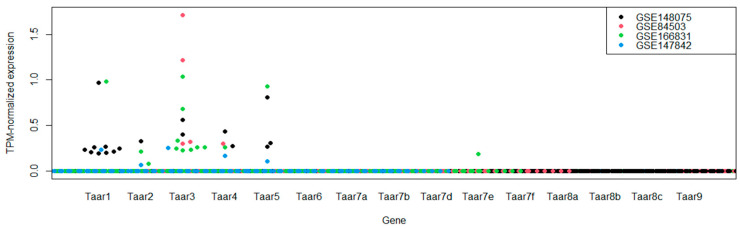
Expression levels of TAARs in whole-tissue mouse hippocampal samples represented in the GEO database.

According to the data obtained, Taar1, Taar2, Taar3, Taar4 and Taar5 are sporadically expressed in mouse hippocampal tissue. In one sample, the Taar7e receptor was also expressed at extremely low levels. The expression patterns of the TAARs in the different datasets appear to be congruent. Expression of Taar3 and Taar4 was detected in all four series in at least one sample per dataset, and Taar1, Taar2 and Taar5 were detected in three datasets (Figure 2). No expression of Taar1, Taar2 or Taar5 was detected in the GSE84503 dataset. It should be noted that, unlike the other three datasets, the dentate gyrus in the samples represented in GSE84503 was trimmed prior to RNA sequencing.

According to Expression Atlas terms, the expression level is below the cut-off if the TPM-normalized level is less than 0.5 TPM [21]. In the analyzed datasets, TAARs are generally expressed at levels below 0.5 TPM. On the other hand, the coherent expression patterns of these receptors in the different sample series studied independently in different institutions suggest that the occurrence of positive expression levels of Taar1–Taar5 in the hippocampal tissues is not a coincidence.

### 3.2. TAARs mRNA Expression in CA1 Pyramidal Cells

Dataset GSE94559 contains data from 12 samples represented by pooled ribosome-associated mRNA from CA1 neurons of 4–6 mice. Six wild-type samples and six samples from *Frn-1* knockout animals were analyzed [22]. No statistically significant differences in TAAR expression levels were found between wild-type and knockout animals.

**Figure 3:**
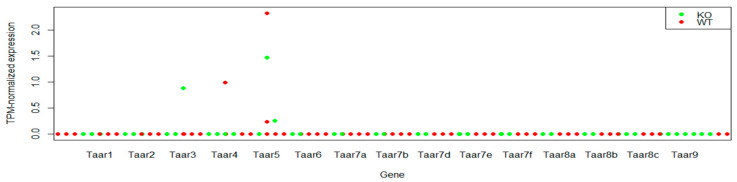
Expression levels of TAARs in mouse hippocampal CA1 pyramidal neurons (GSE94559). KO—Frm1 knockout mice; WT—wild-type control group.

*Frn-1* knockout is the model of fragile X syndrome. Dysfunction of this gene has significant effects on synaptic and structural plasticity in CA1 pyramidal cells [23]. Considering the pronounced effects of *Frn-1* knockout, we analyzed TAARs expression in control and knockout mice separately. Weak Taar5 expression was detected in two wild-type samples and two corresponding knockout mice. Taar3 and Taar4 expression was detected in individual samples in both cases (Figure 3). Thus, the pattern of TAARs expression in mouse hippocampal CA1 pyramidal neurons is not identical to the TAARs expression pattern in whole tissue samples. This result confirms that CA1 pyramidal neurons are involved in the TAARs expression profile in the hippocampus, but other cells also contribute to the described pattern.

### 3.3. Single-Cell RNA-Seq Data

**Figure 4:**
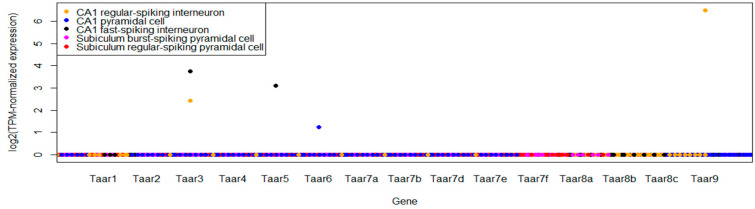
Distribution of TAARs expression in the murine hippocampal cell populations in the dataset GSE75386. Different cell types are macked by color.

Two single-cell datasets were included in the review. In the GSE75386 dataset, cells were collected via a pipette aspiration from the hippocampal slice [24]. Ninety-seven single-cell samples were analyzed. Only in five cells, TAARs mRNA was revealed, including Taar3 expression in two cells and single cases of Taar5, Taar6 or Taar9. Taar3 was expressed in CA1 regular-spiking and C1 fast-spiking neurons, another fast-spiking neuron was Taar5-positive, Taar6 was expressed in a single CA1 pyramidal cell, and Taar9 mRNA was revealed in a single regular-spiking neuron (Figure 4). A notable fact is that neither Taar6 nor Taar9 was expressed in whole-tissue or pooled CA1 neurons mRNA.

**Figure 5:**
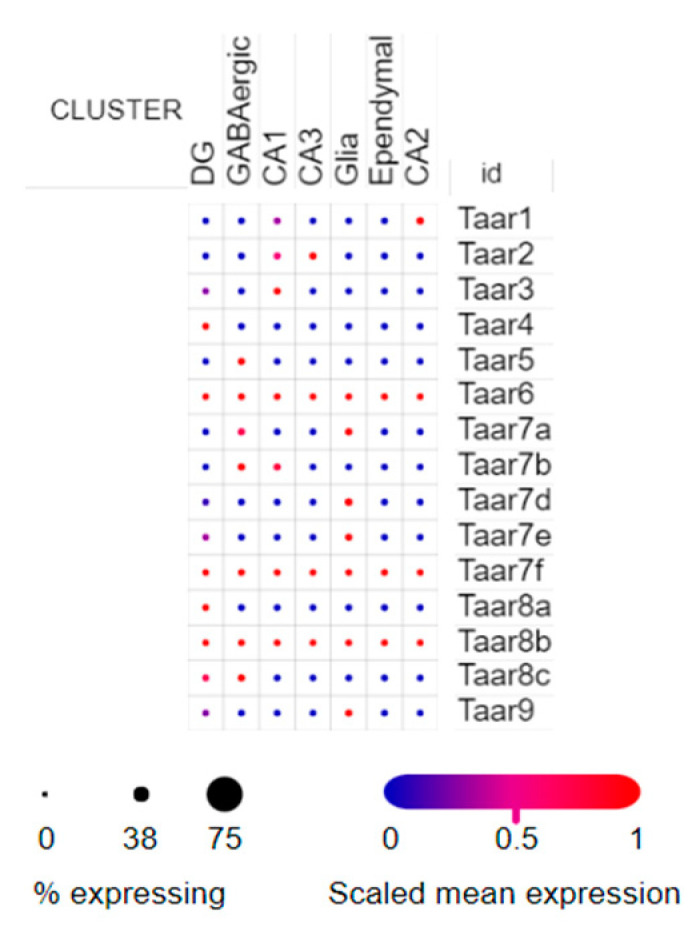
Distribution of TAARs expression in the murine hippocampal cell populations in the SCP1 dataset (picture was generated by the Single Cell Portal interactive interface). Expression levels are marked by color in accordance with the color scale.

A more complex TAAR expression pattern was found in the SCP1 dataset on the Single Cell Portal. This dataset demonstrates the expression in isolated FACS-sorted nuclei, including 1367 single nuclei from hippocampal anatomical subregions (DG, CA1, CA2, and CA3), GABAergic neurons and non-neuronal cells such as glia and ependyma [25]. The samples were obtained from adult mice. All TAARs are expressed in this dataset, and all of them are expressed sporadically, only in several cells. Taar6, Taar7f and Taar8 mRNAs were detected in all cell groups and annotated in the dataset. Taar1, Taar2, Taar3, Taar4, Taar 5, Taar7b, Taar8a and Taar8c expression was demonstrated only in neuronal cells. At the same time, Taar7a, Taar7d, Taar7e and Taar9 were predominantly expressed in the glial cells (Figure 5).

Thus, TAARs expression in the mouse hippocampus seems to be sporadic and low-positive, and its distribution between hippocampal structures and different cell types is heterogeneous. It may be estimated only in deep-sequenced samples. The obtained results do not answer the question whether TAARs expression in the murine hippocampus is constitutive or inducible and context-dependent. At the same time, acquired expression patterns are reproducible and demonstrated in different studies performed independently in several laboratories.

### 3.4. Genotype-Tissue Expression (GTEx) Data for TAARs Expression in the Human Hippocampus

**Figure 6:**
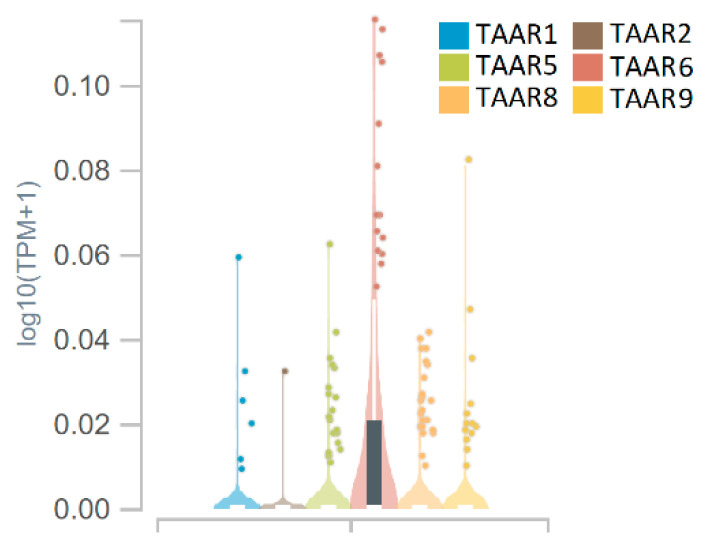
TAAR1, TAAR2, TAAR5, TAAR6, TAAR8 and TAAR9 expression in the human brain structures in male and female in the GTEx Analysis V8 (dbGaP Accession phs000424.v8.p2) dataset. Picture was generated by the GTEx Portal interactive interface.

GTEx Analysis V8 (dbGaP Accession phs000424.v8.p2) dataset demonstrates that TAARs expression pattern in the human hippocampi is not fully congruent to the mouse data. Only TAAR6 expression reaches TPM cut-off 0.1 in the small number of samples in this dataset. At the same time, TAAR6 mRNA is absent in the murine samples. All other TAARs, including TAAR1, TAAR2, TAAR5, TAAR8 and TAAR9, were accidentally expressed in a few samples at the below cut-off levels (Figure 6).

## 4. Discussion

TAARs belong to the class of GPCRs. Humans have 6, and mice have 15 functional TAARs genes. The majority of them (TAAR2–TAAR9) were originally supposed to play a role in sensing innate odors. However, they were also identified in the gastrointestinal tract, heart, kidneys, platelets, the spinal cord and brain [12]. TAAR1 has been shown to play a role in regulating processes such as cognition, nutrition, movement and mood [12,26,27,28]. However, the role of other TAARs in the regulation of the activity of the central nervous system is not yet fully understood, and determining their localization is essential for understanding their neuronal functions. Adult neurogenesis, as one of the mechanisms for maintaining the integrity and plasticity of the central nervous system, is extremely complexly regulated. The indication of the participation of TAARs in this process is based on the data obtained from knockout animals, in which mice lacking TAAR2 [16] and TAAR5 [14,15,17,29] showed increased levels of DCX and PCNA-positive cells in the DG. Furthermore, these mutants have an increased level of brain-derived neurotrophic or glial cell-derived neurotrophic factors, respectively, in the striatum and show an increased number of dopaminergic neurons in the substantia nigra.

We analyzed the open-source data including RNA and scRNA sequencing. The study has some considerable limitations. Sequencing depth in public transcriptomic data is often suboptimal for the evaluation of TAARs expression. TAARs are characterized by an extremely low level of expression; therefore, we used the reading depth with a minimum number of reads in SRA files > 50 million as a criterion for including data in the analysis. Commonly, expression levels in the samples mentioned as TAARs-positive do not reach the cut-off level recommended in the Expression Atlas database [21].

Paying attention to all limitations, we detected an expression of TAAR1, TAAR2, TAAR3, TAAR4 and TAAR5 in RNA sequencing of whole hippocampal tissue from mice. This expression level is extremely low, and it can be characterized as sporadic; in most of the specimens, it remained negative. One of the datasets was obtained from the whole tissue of the hippocampus, but the DG was previously removed from it, and in this case, we did not detect expression of TAAR1, TAAR2 and TAAR5.

Data from single-cell RNA sequencing as well as sequencing of CA1 cells isolated from the mouse hippocampus show that TAAR3 is expressed in these cells, as well as TAAR4. Otherwise, single-cell RNA sequencing data confirmed that TAARs expression in various cells of the hippocampal formation is low and mostly sporadic, wherein TAARs can be detected both in neuronal (TAAR1, TAAR2, TAAR3, TAAR4, TAAR5, TAAR7b, TAAR8a and TAAR8c) and glial (TAAR7f, TAAR7e, TAAR7e and TAAR9) cells. A low level of expression of all functional TAARs was also observed in the human hippocampus. These data are consistent with a previous study, where we found a predominantly limbic pattern of distribution of TAAR5 in the human brain in a similar transcriptomic analysis [30]. A similar conclusion was reached in the transcriptomic analysis performed by an independent group that found the expression of all TAARs, with TAAR5 being the most highly expressed, in the limbic brain areas, including the hippocampus [31].

TAARs are tuned to broad and overlapping sets of ligands (mostly represented by products of amino acid decarboxylation). The most known among them are β-phenylethylamine (PEA), tyramine, tryptamine, octopamine, 3-iodothyronamine (T1AM) and biogenic amines such as dopamine or serotonin [12,32]. The involvement of trace amines and TAARs in hippocampal functioning and adult neurogenesis is yet to be understood. In addition to observations gained in TAAR2 and TAAR5 knockout mice [14,15,16,17,29], there are some indirect lines of evidence on the potential role of trace amines and other TAARs ligands on adult neurogenesis in the SGZ.

It was shown that TAAR1 ligand PEA [10] modulates the BDNF, which plays a critical role in neurogenesis regulation through Raf/ERK1/2 signaling pathway [33]. It is suggested that PEA restores dendritic spine number in the hippocampus in the cortisol-induced depression mouse model by the TAAR1 activation and consequent initiation of BDNF/TrkB/CREB signaling [34,35]. Additionally, PEA restores dendritic spine number in the hippocampus in the cortisol-induced depression mouse model [34]. Likewise, the depletion of monoamine oxidase isoenzyme MAO-B, which preferentially degrades PEA and other trace amines, is associated with enhanced neurogenesis in mice [36]. Another TAAR1 and TAAR5 ligand T1AM [37] increased ERK levels and adult neurogenesis in the hippocampus [38,39,40], while ERK MAP-kinase is the key molecule of BDNF signaling and mediates antidepressant efficacy in humans and animal models of depression [41].

TAARs can be activated by some polyamines. Cadaverine and diamine putrescine binding to TAAR6 and TAAR8 were also studied in silico [42,43]. Additionally, spermidine, spermine and cadaverine binding on TAAR9 was discovered both in silico [44] and in vitro [45,46]. Moreover, TAAR1 also mediates the response to spermidine or cadaverine that has been shown in the choroid plexus papilloma HIBCPP cells [47]. Spermidine, spermine and putrescine distribution in the brain, and particularly in the hippocampus, is heterogeneous [48,49]. Evidently, these molecules are involved in a plethora of biological processes due to their ability to bind DNA, RNA, protein kinases/phosphatases, enzymes, and ionic channels. They are released in the synapses upon K^+^ depolarization [50] and were detected in the synaptosomes [51]. Acute depletion of putrescine in the hippocampus leads to anxiety-like behavior and impairs memory. A higher level of spermidine in DG is associated with decreased anxiety, whereas spermine in DG had a completely opposite pattern [52]. Putrescine stimulates neural progenitor proliferation. Its depletion leads to the disorder of the neural progenitor cell cycle [53]. Spermine reverses the LPS-induced decrease in mature BDNF levels in the hippocampus in mice [54]. Interestingly, polyamine levels are altered after traumatic brain injury [55] and stroke [56], conditions affecting adult neurogenesis [57]. The contribution of TAARs in polyamine-dependent processes remains unknown. It should be noted that polyamines have multiple molecular targets and could also alter the activity of NMDA, AMPA, TRPV1 and kainate receptors [50].

It is proposed that TAAR6 might represent one of the endogenous hallucinogen receptors for N, N-dimethyltryptamine (DMT) [58]. DMT is also able to activate TAAR1 at high concentrations [58]. This ligand also binds subtypes 1a and 2b of the serotonin receptor (5-HT) and non-serotonergic receptors, such as the sigma-1 receptor S1R [59]. DMT treatment activates the subgranular neurogenic niche regulating the proliferation of neural stem cells and the migration of neuroblasts and promoting the generation of new neurons in the hippocampus. Pro-neurogenic effect of DMT depends on its binding by S1R [59]. The potential involvement of TAAR1 or TAAR6 in DMT-dependent neurogenesis activation has yet to be studied.

Monoamine neurotransmitters, such as norepinephrine, dopamine and serotonin (5-HT), are involved in the adult neurogenesis regulation via activation of their respective GPCRs [8,9]. Previously, it was shown that TAARs may physically interact with monoaminergic receptors and modulate their function [12]. The most studied of such interactions is the dimerization of TAAR1 with dopamine receptor D2R. After the heterodimerization with TAAR1, D2R activity shifts from the β-arrestin-2 signaling pathway to G_i_ activation [12,17,60]. The role of D2R-mediated dopamine signaling in neurogenesis is predominantly inhibitory, and D2R antagonist haloperidol activates neuronal stem cell proliferation [9]. However, this effect is context-dependent and has been shown in prenatally stressed mice, among which selective agonist of D2R receptors ropinirole restores neurogenesis and the number of cells in the hippocampus [61].

TAAR1 also has the potential to interact with the 5-HT1b serotonin receptor, which is considered to be involved in newborn cell survival in the adult dentate gyrus [9,62,63]. Thus, the 5-HT effect on 5-HT1b on this heterodimer becomes less pronounced than in its homodimeric form. Additionally, 3-T1AM initiates G_s_ activation after the binding on TAAR1/5-HT1b heterodimer instead of Gi/o signaling activation after its recognition by homodimeric 5-HT1b [62]. 5-HT shows a complex effect on adult neurogenesis depending on its concentration and receptors pattern [64]. Altogether, TAAR1 forming dimer at least with the 5-HT1b receptor may also modulate this context-dependent 5-HT function.

Norepinephrine regulates latent neural stem cell activity and adult hippocampal neurogenesis through α2- and β-adrenergic receptors. For instance, stimulation of α2-adrenergic receptors inhibits hippocampal precursor activity and decreases hippocampal neurogenesis, and stimulation of β-adrenergic receptors activates the quiescent precursor pool and enhances their proliferation in the adult hippocampus [8,65]. Therefore, TAAR1 forms hetero-oligomers with α2A-adrenoreceptors, and these oligomers bind norepinephrine, which activates G protein-coupled ADRA2A-dependent cAMP down-regulation in the presence of T1AM [37].

The amount of data indicating the existence of a relationship between the system of trace amines, their receptors and neurogenesis is constantly increasing. It should be noted that while the majority of current data points out the role of TAARs in adult neurogenesis, an intriguing possibility of involvement of TAARs in the neurogenesis during development remains essentially unexplored and requires careful investigation. Here, we demonstrated that certain TAARs are expressed in various types of cells in mouse and human hippocampal formation at a low but detectable level. Furthermore, we found the difference in the representation of different types of TAARs between mouse and human samples. At the moment, it is difficult to explain this difference because the number of TAARs in mice is greater than in humans and their neuronal functions, which are not yet fully understood, may not be identical. Nevertheless, the present observations provide additional support to the data gained in knockout mice that indicate an important role of TAARs in the regulation of adult neurogenesis. Further mechanistic studies are warranted to explore if such regulation can occur directly via TAAR-related signaling events or indirectly through an additional involvement of other monoaminergic mediators and GPCRs.

## Figures and Tables

**Table 1:** RNAseq datasets included in the review.

Accession Number	Title	Number of Samples	Experiment Design
Whole-tissue data			
GSE84503	Activity-dependent regulation of alternative cleavage and polyadenylation (APA) during hippocampal long-term potentiation (LTP) [RNA-Seq]	6 control samples/6 experimental samples	Acute hippocampal slices after chemically induced long-term potentiation induction, potentiated slices and time-matched control slices were studied 1 hr and 3 hr after the intervention
GSE147842	Adult mouse hippocampal transcriptome changes associated with long-term behavioral and metabolic effects of gestational air pollution toxicity	10 control samples/10 experimental samples	Pregnant dams were exposed to urban derived nanosized particulate matter during the gestational period. The effects were studied in adults
GSE148075	Wild mice with different social network sizes vary in brain gene expression	14 in the “high gregariousness” group and 15 in “low gregariousness” group	Dataset from three brain regions (hypothalamus, prefrontal cortex and hippocampus, only hippocampal samples were included in the review) from wild mice presenting large or small social network sizes
GSE166831	Altered hippocampal transcriptome dynamics following sleep deprivation	9 control samples/9 experimental samples	Whole hippocampus RNA profiles of mice who were either sleep-deprived for 5 h or left undisturbed
Cell fractions data			
GSE94559	Hippocampus CA1 pyramidal cells Transcriptomic profile in WT and Fmr1 KO mice, using Wfs1-CreERT2:RiboTag:Frm1 knockout and wild-type mice	6 control samples/6 experimental samples	Pairwise comparison of CA1 pyramidal cells in wild-type and Fmr1 KO mice
Single-cell data			
GSE75386	Single-cell RNAseq of electrophysiologically characterized neurons of the hippocampus	93 single-cell samples	CA1 cholecystokinin, parvalbumin and pyramidal neurons, as well as subiculum burst and regular firing pyramidal neurons, were studied
SCP1	Single nucleus RNA-seq of cell diversity in the adult mouse hippocampus	1367 single nuclei	Nuclei from hippocampal anatomical sub-regions DG, CA1, CA2, CA3 and lowly abundant GABAergic neurons were analyzed

## Data Availability

Not applicable.
